# 自噬与铁死亡在肺癌发生发展及治疗中的研究进展

**DOI:** 10.3779/j.issn.1009-3419.2025.102.41

**Published:** 2025-10-20

**Authors:** Chengqi JIANG, Xueping CUI, Li ZHENG, Chengkun DENG, Ruoshan HUANG, Bo HOU, Junfeng WANG

**Affiliations:** 655000 曲靖，云南省曲靖中心医院胸外科; Department of Thoracic Surgery, Qujing Central Hospital, Qujing 655000, China

**Keywords:** 肺肿瘤, 自噬, 铁死亡, 信号通路, 非编码RNA, Lung neoplasms, Autophagy, Ferroptosis, Signaling pathway, Non-coding RNA

## Abstract

肺癌是严重威胁人类健康的恶性肿瘤，其发病机制复杂多样。本文系统综述自噬与铁死亡相关的信号通路和关键调控因子及其相关机制，包括核受体共激活因子4（nuclear receptor coactivator 4, NCOA4）介导的铁蛋白自噬-铁死亡轴、线粒体自噬、脂滴自噬、生物钟自噬、分子伴侣介导的自噬等，阐述肿瘤微环境与非编码RNA对肺癌的自噬-铁死亡作用。综述进一步阐述现代药物及中药活性成分通过靶向自噬和铁死亡改善肺癌的潜力，提出靶向二者的交互通路可为肺癌的治疗提供新策略。

## Abstract (en)

Lung cancer remains a life-threatening malignancy with complex pathogenesis. This paper provides a systematic review of autophagy and ferroptosis-related signaling pathways, key regulatory factors, and their associated mechanisms, including the nuclear receptor coactivator 4 (NCOA4)-mediated ferritin autophagy-ferroptosis axis, mitochondrial autophagy, lipid droplet autophagy, circadian autophagy, chaperone-mediated autophagy, *etc.*. The review elucidates the roles of the tumor microenvironment and non-coding RNAs in autophagy-ferroptosis processes in lung cancer. Furthermore, it explores the potential of modern drugs and active components from traditional Chinese medicine to improve lung cancer outcomes by targeting autophagy and ferroptosis, proposing that targeting their interactive pathways could offer novel therapeutic strategies for lung cancer.

肺癌是目前全球发病率和死亡率均排名第一的恶性肿瘤，已连续十年位居全球癌症死亡率首位。世界卫生组织最新数据^[1]^显示，2020年全球肺癌死亡病例高达180万例，占癌症死亡总数的18%。其中小细胞肺癌（small cell lung cancer, SCLC）患者占10%-15%，而非小细胞肺癌（non-small cell lung cancer, NSCLC）患者占80%-85%。尽管在诊断和治疗方面取得了显著进展，但肺癌的5年生存率仍较低，仅为20%左右^[2]^。因此，深入阐明肺癌发生发展的关键分子机制、识别潜在作用靶点、建立新型治疗策略仍然是当前研究亟需面对的核心挑战。

近年来，随着探索的不断深入，研究发现自噬和铁死亡通过相互影响共同参与多种疾病的发生与发展，也为肺癌的诊治提供了新的探索方向。自噬是一种高度保守的细胞自我降解过程，主要通过腺苷酸激活蛋白激酶（adenosine monophosphate-activated protein kinase, AMPK）/哺乳动物雷帕霉素靶蛋白（mammalian target of rapamycin, mTOR）等信号通路来回收降解受损的细胞器、蛋白质和生物大分子，维持细胞内环境稳态^[3]^。而铁死亡是一种自噬依赖性细胞死亡，是由脂质过氧化所触发的细胞死亡，该脂质过氧化过程由铁催化启动，具体涉及脂氧合酶介导的酶促机制与芬顿反应介导的非酶促机制^[4]^。其中，铁积累与脂质过氧化是驱动铁死亡发生发展的两个关键核心介质。自噬对铁死亡具有重要调控作用，其可通过对特定细胞器或蛋白质的选择性溶酶体降解，调控细胞内铁积累水平与脂质过氧化程度，进而影响铁死亡进程。同样，铁死亡也可以调节自噬溶酶体（autophagic lysosomes, AL）的形成，从而影响自噬过程，形成二者间的相互调控关系^[5]^。基于此，本文重点梳理了自噬和铁死亡与肺癌之间存在的联系，并探讨其作为临床治疗靶点的潜在价值，以期为肺癌防治策略提供创新性干预思路。

## 1 自噬与铁死亡

### 1.1 自噬的主要机制

自噬受多种信号分子调控，包括自噬相关基因（autophagy-associated genes, ATGs）及磷脂酰肌醇3-激酶（phosphatidylinositol 3-kinase, PI3K）等，并通过AMPK/mTOR和缺氧诱导因子（hypoxia-inducible factor, HIF）等通路调节。自噬包括启动、成核、延伸、成熟、融合与降解六个阶段（图1）。启动时，饥饿条件下AMPK抑制mTOR，激活Unc-51样自噬激活激酶1（Unc-51 like autophagy activating kinase 1, ULK1）复合物启动自噬；成核中ULK1激活Beclin1-液泡分选蛋白34（vacuolar protein sorting 34, Vps34）复合物，产生磷脂酰肌醇3,4,5-三磷酸（phosphatidylinositol 3,4,5-trisphosphate, PIP3）促进吞噬泡形成。延伸阶段涉及ATG7激活ATG12，与ATG5、ATG16形成复合物，同时前体蛋白前体微管相关蛋白1轻链3（pro-microtubule-associated protein 1 light chain 3, pro-LC3）经ATG4等加工为LC3-II；成熟后自噬体与溶酶体在溶酶体相关膜蛋白2（Ras-associated binding protein 2, Rab2）等蛋白介导下融合形成AL，最终降解内容物并释放回胞质^[3]^。自噬通过多分子协同、多阶段衔接的精密机制，实现异常组分的高效清除与资源再利用，维持细胞稳态。

**图1:**
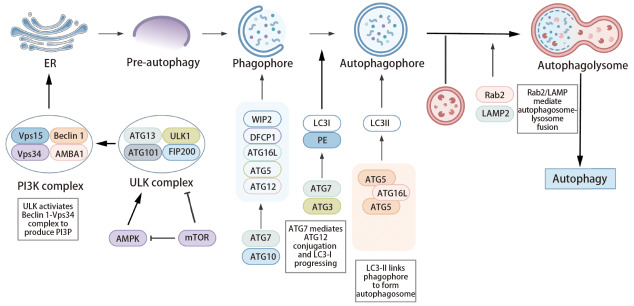
自噬的主要机制

### 1.2 铁死亡的主要机制

铁死亡是一种铁依赖性的程序性死亡，现已确定其属于自噬机制的自噬依赖型细胞死亡的一种形式。正常情况下，细胞主要通过转铁蛋白受体1（transferrin receptor 1, TFR1）摄取铁，部分储存于铁蛋白，部分参与代谢。铁死亡的发生机制可大致分为外源性或转运蛋白依赖途径与酶调节途径（图2）。核受体共激活因子4（nuclear receptor coactivator 4, NCOA4）作为铁蛋白自噬的关键受体，可特异性识别并结合铁蛋白，引导至溶酶体降解，释放Fe³^+^；Fe³^+^还原为Fe²^+^后增加细胞内游离铁，通过芬顿反应产生活性氧（reactive oxygen species, ROS），如羟基自由基攻击多不饱和脂肪酸（polyunsaturated fatty acid, PUFA），引发脂质过氧化链式反应，生成脂质过氧化物（phospholipid hydroperoxides, L-OOH）。谷胱甘肽过氧化物酶4（glutathione peroxidase 4, GPX4）是细胞内抑制脂质过氧化的关键酶，利用还原型谷胱甘肽（glutathione, GSH）将L-OOH还原为无害的脂质醇（lipid alcohol, L-OH）。当GPX4活性丧失或表达降低会导致L-OOH积累，诱发铁死亡。此外，system Xc-介导的胱氨酸摄取和GSH合成以及花生四烯酸代谢等通路也参与调控铁死亡^[6-8]^。铁死亡的核心是铁代谢异常与抗氧化防御失效，其调控网络涉及多代谢途径，与癌症有关并为治疗提供潜在靶点。

**图2:**
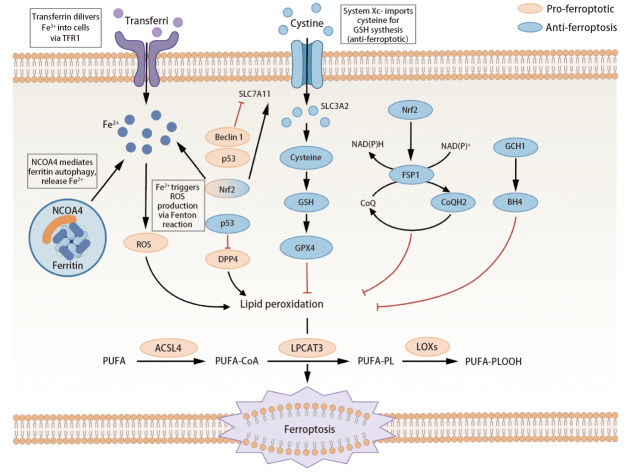
铁死亡的主要机制

## 2 自噬与铁死亡在肺癌发生发展中的作用

在肿瘤发生早期，自噬主要通过清除受损细胞器和异常蛋白质维持细胞内环境稳态，从而发挥肿瘤抑制作用。随着肿瘤进展至晚期，自噬功能发生转变，通过帮助癌细胞适应缺氧、营养缺乏及药物治疗等应激压力，转而促进肿瘤存活。此时，自噬对铁死亡的调控也变得更为复杂，可根据具体肿瘤微环境（tumor microenvironment, TME）条件双向调节细胞的发展。近年研究^[9,10]^逐渐揭示自噬与铁死亡在肺癌进程中并非孤立存在，而是互为关联。值得注意的是，自噬在特定条件下能够扮演铁死亡的驱动角色，这种被称为“自噬依赖性铁死亡”的过程，涵盖了铁蛋白自噬、线粒体自噬等多种路径（图3）。

**图3:**
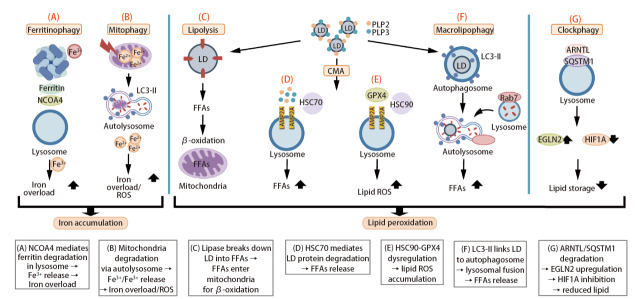
自噬与铁死亡作用机制示意图

### 2.1 NCOA4介导的铁蛋白自噬-铁死亡轴

铁蛋白自噬是指通过自噬途径选择性降解铁蛋白，以调节细胞内铁的稳态和铁依赖性细胞死亡，如铁死亡过程。在铁缺乏条件下，细胞需要通过降解铁蛋白释放储存的铁离子以满足代谢需求，此过程主要依赖于自噬途径。作为铁蛋白自噬的关键受体，NCOA4可特异性识别并结合铁蛋白，引导至溶酶体降解，释放Fe³^+^；Fe³^+^还原为Fe²^+^后增加细胞内游离铁，通过芬顿反应产生ROS，引发脂质过氧化，从而促进铁死亡。在肺腺癌中，肿瘤抑制因子Par-4可通过激活NCOA4依赖的铁蛋白自噬，增强铁死亡，限制肿瘤进展^[11]^。相反，致癌基因*c-Myc*通过下调NCOA4表达，抑制铁蛋白自噬，减少细胞内游离铁蓄积与铁死亡发生，进而促进癌细胞增殖与免疫逃逸^[12]^。此外，肺腺癌患者中NCOA4的低表达通常预示着更差的总生存期^[13]^。当核糖体核酸蛋白复合物I亚基ζ1（coatomer protein complex I subunit zeta 1, COPZ1）敲低时，会解除对NCOA4的抑制，增强铁蛋白自噬，促进铁死亡，从而抑制肺腺癌肿瘤的生长^[14]^。理论上，对于NCOA4低表达的肿瘤，可能存在铁死亡过程的抑制，使其对氧化应激不敏感。此时，若使用铁死亡诱导剂，可能特异性地杀伤这类肿瘤细胞，考虑将铁死亡诱导剂与现有的免疫检查点抑制剂（immune checkpoint inhibitors, ICIs）等联合使用，以期实现更好的治疗效果。

### 2.2 线粒体自噬与铁死亡之间的作用

近年来，线粒体自噬与铁死亡之间的相互作用逐渐受到关注。在肺癌中，线粒体自噬通过选择性清除受损的线粒体，抑制ROS的过度积累和铁离子的释放，从而在一定程度上保护肿瘤细胞免受铁死亡的损伤。研究^[15]^表明，线粒体自噬缺陷会增强肺癌细胞对铁死亡的敏感性，促进肿瘤细胞死亡，反之通过促进线粒体自噬可延缓病理状态的恶化。在铁过载的早期阶段，细胞将过量铁暂存于线粒体中，以缓解胞质游离铁激增，随后线粒体自噬把铁隔离在吞噬体中，短暂削弱芬顿反应和ROS产生。然而，持续铁过载会造成线粒体损伤，反过来激活更广泛的线粒体自噬，导致被隔离的铁、ROS和过氧化脂质大量外泄，从而激活各种ROS诱导的细胞死亡途径，包括铁死亡^[16]^。针对这一过程的调控，研究发现低氧诱导因子1A（hypoxia-inducible factor 1 alpha, HIF1A）抑制剂BAY 87-2243在肺癌模型中能够抑制线粒体呼吸链复合物I，诱导线粒体膜电位丧失并刺激线粒体自噬，最终增加铁死亡的发生，而敲低关键线粒体自噬基因*PINK1*则可抑制BAY 87-2243诱导的铁死亡^[17]^。此外，在肺癌细胞中阻断PINK1/Parkin通路可提高细胞内铁离子浓度，减少线粒体自噬，增加脂质过氧化，从而增加了细胞对铁死亡的脆弱性^[18]^。肿瘤蛋白53（tumor protein 53, p53）和核转录因子红系2相关因子2（nuclear factor erythroid 2-related factor 2, Nrf2）作为铁死亡相关信号通路中的两种分子，也参与调控线粒体自噬^[19]^。同时，p53还能影响铁死亡敏感性，并调控铁死亡相关基因的表达^[20]^。作为关键抗氧化转录因子，Nrf2调控细胞内铁代谢及抗氧化酶相关基因的表达。铁死亡过程中Nrf2活性可能发生改变，进而影响线粒体自噬进程^[21]^。多种信号通路的相互作用揭示了肺癌细胞中线粒体自噬与铁死亡的精细调控机制。

### 2.3 脂滴自噬与铁死亡之间的作用

脂滴自噬是脂滴在溶酶体中降解的过程，其核心机制涉及多个关键蛋白：脂滴表面的外壳蛋白（Perilipin 2和Perilipin 3）被热休克同源蛋白70（heat shock cognate protein 70, HSC70）和溶酶体相关膜蛋白2A（lysosomal-associated membrane protein 2A, LAMP2A）识别，进而被LC3阳性自噬膜吞噬并运往溶酶体，最终由溶酶体酸性脂肪酶分解。此过程通过增强脂质降解与释放游离脂肪酸，维持细胞内脂质稳态。脂滴在肺癌细胞中对铁死亡的调控作用呈现出复杂的双重性。一方面，脂滴的分解理论上可通过释放游离多不饱和脂肪酸（polyunsaturated fatty acid, PUFA）而促进脂质过氧化和铁死亡。但另一方面，研究^[22]^揭示，在肺癌的发生发展中，脂滴更可能扮演着一种细胞自我保护的角色。例如，在肺腺癌中，当肿瘤细胞面临铁死亡压力时，会通过上调DGAT1/2来加速甘油三酯的合成并促进脂滴的形成^[22]^。因此，未来针对脂滴自噬与铁死亡在肺癌中调控关系的探索，应着重考察干预脂滴稳定性对肺癌细胞耐药性的影响。基于现有发现，诱导脂滴自噬以分解其储存的脂质，可能通过增加细胞内游离PUFA水平，增强耐药肺癌细胞对铁死亡的敏感性。这一方向尚需实验研究进一步验证。

### 2.4 生物钟自噬与铁死亡之间的作用

哺乳动物的昼夜节律由中央时钟和外周时钟共同调控，中央时钟位于下丘脑视交叉上核（suprachiasmatic nucleus, SCN），主要受光信号等外界环境因素调控；外周时钟则广泛存在于几乎所有组织和细胞中，由SCN通过激素、代谢因子及体温等全身性变化进行调节。以时钟生物节律调节因子（clock circadian regulator, CLOCK）和芳香烃受体核转位样蛋白（aryl hydrocarbon receptor nuclear translocator like, ARNTL）异源二聚体为核心的转录翻译反馈调节回路（transcription-translation feedback loop, TTFL），是中央时钟与外周时钟实现昼夜节律调控的关键分子机制^[23]^。近年来，生物钟自噬与细胞稳态维持之间的相互作用逐渐受到关注。生物钟自噬通过节律性地调控自噬活性，清除受损细胞器和蛋白质，从而在能量代谢和应激抵抗中维持细胞的周期性平衡^[24]^。在肺腺癌中，ARNTL2受到抑制可促进肺腺癌铁死亡、抑制上皮间质转化、细胞增殖、迁移和侵袭^[25]^。Yang等^[26]^鉴定出SQSTM1作为自噬性ARNTL降解的运货受体，并进一步揭示了其在肺癌中的下游机制，ARNTL通过抑制EGLN2转录来抑制铁死亡，从而激活促生存转录因子HIF1A。阻断ARNTL降解会减弱铁死亡性肿瘤细胞死亡，而破坏HIF1A稳定性则会促进该过程。因此，ARNTL有望成为治疗肺癌新的靶点。靶向调控ARNTL的稳定性或抑制其降解机制，可能通过减轻氧化应激和铁死亡为肺癌的治疗提供新方向。然而，目前关于生物钟自噬与肺癌的具体分子机制仍需深入探索，未来需结合临床转化研究验证其治疗潜力。

### 2.5 分子伴侣介导的自噬（chaperone-mediated autophagy, CMA）与铁死亡之间的作用

CMA主要通过降解抗氧化防御系统中的关键蛋白来影响脂质过氧化进程。研究^[27]^表明，在Erastin诱导的铁死亡过程中，热休克蛋白90（heat shock protein 90, HSP90）可通过CMA途径介导GPX4的降解。GPX4作为细胞内重要的抗氧化酶，其本身也可通过自噬-溶酶体途径被降解，直接削弱细胞清除L-OOH的能力，从而显著增强细胞对铁死亡的敏感性。在NSCLC中，靶向抑制CMA（通过敲低LAMP2A）不仅能够稳定GPX4蛋白水平，减少铁死亡的发生，还能有效抑制肿瘤细胞的增殖、克隆形成能力，并增强其对化疗药物的敏感性，进一步的动物实验^[28]^证实，LAMP2A敲低可抑制移植瘤的体内生长，并增强荷瘤小鼠对顺铂的治疗反应。此外，在NSCLC中CMA水平高于正常状态，遗传学干预阻断CMA后，可显著抑制癌细胞的生长和转移^[29]^。在小鼠模型中，NSCLC异种移植中的*LAMP2A*敲低作用使肿瘤细胞对化疗敏感；在人体组织中，无反应患者的LAMP2A蛋白表达水平更高^[28]^。这些事实在化疗中可能具有潜在价值，例如作为预测患者治疗反应的生物标志物，或作为化疗耐药性肿瘤的治疗选择。目前，CMA相关分子在人类肿瘤中的表达数据仍很有限，未来需要更多研究来测定肿瘤CMA水平，从而验证上述假说。

## 3 TME与非编码RNA（non-coding RNA, ncRNA）对肺癌的自噬-铁死亡的作用

### 3.1 TME对自噬和铁死亡的影响

TME由癌细胞、免疫细胞、基质细胞、血管细胞、细胞外基质及各种细胞因子共同构成，是肿瘤发生、发展、转移及治疗反应的重要调控因素。在肺癌中，TME的异质性和免疫抑制特性是驱动肿瘤进展和治疗耐药的关键。近年来，研究^[5]^表明，自噬与铁死亡作为两种重要的细胞程序性死亡形式，在TME中呈现复杂的相互作用，影响肿瘤细胞的存活、免疫逃逸及治疗敏感性。本文提出一种系统性研究框架：首先阐明自噬相关铁死亡的机制，继而探讨其在TME中的作用。信号转导与转录激活因子3（signal transducer and activator of transcription 3, STAT3）介导的溶酶体膜通透性改变，对铁死亡至关重要。STAT3可促进肿瘤细胞中血管内皮生长因子（vascular endothelial growth factor, VEGF）、白介素-6（interleukin 6, IL-6）和IL-10等免疫抑制因子的表达，这些肿瘤源性物质本身即是STAT3激活剂，可传递至TME，进而增强包括癌相关成纤维细胞（cancer-associated fibroblast, CAF）在内的多种免疫细胞亚群中的STAT3活化。在肺癌模型中，STAT3的持续激活与调节性T细胞的免疫抑制功能及CD8^+ ^T细胞的功能耗竭密切关联^[30]^。此外，STAT3过度激活在树突状细胞（dendritic cell, DC）成熟过程中发挥关键作用。DC对启动T细胞抗肿瘤反应不可或缺，而未成熟DC则诱导免疫耐受。肿瘤细胞中STAT3过度激活可抑制IL-12和肿瘤坏死因子-α（tumor necrosis factor-alpha, TNFα）表达，进而降低DC中B细胞淋巴瘤-2蛋白（B-cell lymphoma 2, Bcl-2）的表达。由于未成熟DC无法激活T细胞，在免疫或肿瘤暴露后，STAT3耗竭造血系统的实验小鼠中，干扰STAT3的CD8^+ ^T细胞可产生更多特异性干扰素-γ（interferon-gamma, IFNγ）^[31]^。在NSCLC中，肿瘤细胞可通过上调二酰基甘油O-酰基转移酶1/2（diacylglycerol O-acyltransferase 1/2, DGAT1/2），将脂质以脂滴形式储存并分泌氧化甘油三酯等脂质至胞外，直接抑制CD8^+ ^T细胞的抗肿瘤功能，从而形成免疫抑制性TME^[22]^。近期研究^[32]^进一步揭示，肺癌TME中的缺氧条件可通过持续激活CD8^+ ^T细胞中的激活转录因子4（activating transcription factor 4, ATF4），诱导线粒体氧化应激及细胞死亡，最终导致CD8^+ ^T细胞功能耗竭并削弱程序性死亡受体1（programmed cell death protein 1, PD-1）抑制剂的疗效。

高迁移率族盒蛋白B1（high mobility group box 1, HMGB1）在促进自噬过程中发挥位置依赖性作用。细胞膜上的HMGB1可与BECN1结合诱导自噬；细胞外HMGB1通过激活PI3K诱导自噬。作为损伤相关的分子模式，细胞外HMGB1在RAS选择性致死化合物3（Ras-selective lethal 3, RSL3）和弹性蛋白诱导的体外铁死亡过程中可引发炎症与免疫反应^[33]^。尤其值得注意的是，HMGB1作为免疫原性细胞死亡（immunogenic cell death, ICD）的重要标志物，能促进DC成熟，并向细胞毒性T淋巴细胞（cytotoxic T lymphocytes, CTL）呈递抗原以激活CTL清除异常细胞。近期实验^[34]^证实，CD8^+ ^T细胞通过调控自噬与铁死亡变化介导抗肿瘤免疫，并与TME相互作用以直接调控抗肿瘤免疫反应。在肺癌中，基于铁死亡诱导的纳米治疗策略能够通过引发肿瘤细胞铁死亡，释放HMGB1，从而促进DC成熟并增强CD8^+ ^T细胞的抗肿瘤免疫应答，最终逆转免疫抑制微环境^[35]^。此外，在NSCLC中，BECN1激活肽Tat-beclin 1可通过促进BECN1与SLC7A11的结合，抑制System Xc-功能，诱导铁死亡，并显著抑制肿瘤生长^[36]^。综上，自噬与铁死亡通过调控肺癌细胞死亡与TME免疫状态，进而影响肿瘤进程。深入阐释二者在肺癌TME中的共同作用，不仅为理解其耐药机制提供新视角，也为开发靶向诱导ICD的联合治疗策略奠定了理论基础。

### 3.2 ncRNA对自噬和铁死亡调控作用

ncRNA作为基因表达调控的重要分子，近年来在细胞自噬和铁死亡两种程序性细胞死亡形式中的调控作用逐渐被揭示。研究^[37,38]^表明，ncRNA通过调控自噬和铁死亡的关键分子和信号通路，参与肿瘤细胞的生存、增殖、转移及药物耐受等过程，揭示了ncRNA介导的自噬与铁死亡的复杂网络。

在肺癌进展过程中，异常调控的非编码ncRNA与自噬作为关键调控因子，通过转录、转录后及翻译后水平调控相关信号通路，从而改变肺癌的多种恶性行为及治疗反应。miRNAs可通过促进或者抑制自噬作为肺癌的促死亡因子。Guo等^[39]^报道，miR-384通过下调胶原α-1（X）链水平，抑制细胞增殖，促进NSCLC细胞凋亡和自噬。自噬相关的长链非编码RNA（long noncoding RNA, lncRNA）也可作为肺癌的启动子和抑制因子。Wu等^[40]^报道，LINC01279在肺癌细胞中高表达，抑制LINC01279功能可减少NSCLC细胞异种移植肿瘤的生长。进一步研究发现，敲低*LINC01279*或*SIN3A*可激活NSCLC细胞的自噬和凋亡。LncRNAs可以通过抑制自噬来抑制肺癌。例如Gupta等^[41]^报道，靶向lncRNADLX6-AS1增强miR-16活性，诱导自噬和细胞凋亡，同时通过miR-16海绵调节BMI1，从而抑制肺癌生长。与传统的线性RNA不同，环状RNA（circular RNA, circRNA）具有闭环结构，使其对RNA核酸外切酶具有抗性。Lu等^[42]^报道，Hsa_circ_0096157通过自噬激活促进NSCLC生长。此外，新型的ncRNA，如vault RNA 1-1，可直接与自噬受体蛋白p62结合，改变其功能，体现了ncRNA在自噬调控中的多样化机制^[43]^。

同样，miRNA、lncRNA和circRNA不仅可以调控铁死亡相关基因的表达，还能影响铁死亡的信号通路，对肿瘤细胞的药物敏感性和生存状态产生影响。外泌体miR-4443可以通过甲基转移酶3（methyltransferase-like 3, METTL3）、铁死亡抑制蛋白1（ferroptosis suppressor protein 1, FSP1）诱导的铁死亡增加顺铂耐药性，从而降低NSCLC的治疗效果^[44]^。Deng等^[45]^证明miR-324-3p可以通过诱导GPX介导的肺腺癌细胞铁死亡来降低顺铂耐药性。另一项研究^[46]^发现，通过miR-365a-3p/Nrf2被叶酸修饰的脂质体纳米颗粒包围的lncRNA MT1DP可以提高对铁死亡的敏感性，并可能成为NSCLC的新治疗方法。此外，与铁死亡相关的lncRNA已被确定为肺腺癌的预后生物标志物的有lncRNA C20orf197、lncRNA ARHGEF26-AS1和lncRNA LINC00324等^[47]^。circRNAs在NSCLC中起作用，例如circDTL通过miR-1287-5p/GPX4通路减少癌细胞的铁死亡^[48]^。

在肺癌进展中，ncRNA通过调控自噬和铁死亡的作用，共同影响肿瘤的增殖、侵袭、转移以及对治疗的响应。例如，肺腺癌中lncRNA YTHDF3-DT通过miR-301a-3p/INHBA轴调节自噬依赖的铁死亡，促进肿瘤细胞存活，提示其为潜在的治疗靶点^[49]^。此外，ncRNA还通过调控自噬和铁死亡信号通路参与肺癌的药物耐受性和放疗抗性，揭示了调节这两种细胞死亡形式的ncRNA在肺癌治疗中的重要性^[50]^。

## 4 靶向自噬-铁死亡轴的肺癌治疗策略

### 4.1 靶向自噬的治疗方法

自噬在肺癌治疗中扮演着双重角色：激活细胞毒性自噬可诱导肿瘤细胞死亡，而抑制保护性自噬则能削弱肿瘤细胞的应激耐受，进而促进凋亡并克服耐药性。目前，靶向治疗策略主要围绕调控PI3K/Akt/mTOR、AMPK/mTOR、MAPK等核心通路及相关自噬蛋白展开。

众多天然小分子中，生物碱、多酚、甾体等在临床前研究中显示出通过上述通路有效诱导肺癌细胞自噬性死亡或凋亡的潜力。生物碱类中，如槲皮素可通过抑制PI3K/Akt/mTOR通路诱导自噬和凋亡^[51]^；多酚类中，如姜黄素常通过激活AMPK/mTOR通路上调自噬标志物LC3-II并抑制细胞增殖^[52]^。在自噬抑制剂方面，羟氯喹可通过阻断自噬体-溶酶体融合抑制保护性自噬，从而增强化疗敏感性^[53]^。盐酸小檗胺等生物碱通过激活ROS/MAPK通路，提高Beclin 1、LC3-II及ROS水平，导致肺癌细胞中引发自噬与凋亡^[54]^。然而，该领域在临床转化中面临显著挑战。多数研究仍停留在体外阶段，缺乏系统的体内验证；天然药物普遍存在生物利用度低、水溶性差等药学瓶颈；而纳米递送系统的靶向效率与生物安全性仍是制约因素。临床层面，羟基氯喹作为自噬抑制剂的临床试验未能显著改善患者预后，且不良反应增加，提示自噬通路存在复杂的代偿机制，单一靶点干预策略的疗效有限。此外，在自噬抑制剂中，ULK1/2抑制剂DCC-3116目前正在进行的I/II期临床试验（NCT04892017），旨在治疗患有RAS/MAPK通路突变的晚期或转移性实体肿瘤患者，包括*KRAS *G12C突变NSCLC患者，但现在尚未披露初步疗效数据^[55]^。因此，未来研究需致力于开发多靶点协同策略、优化药物递送系统并探索预测性生物标志物，以提升靶向自噬治疗的精准性与临床成功率。

### 4.2 诱导铁死亡的治疗方法

诱导肺癌细胞发生铁死亡是一种具有潜力的治疗策略。目前，已有多种铁死亡诱导剂被研发并应用于肺癌研究中。Liang等^[56]^发现，PRLX93936通过增加ROS、过氧化和Fe^2+^水平，同时通过Nrf2/Kelch样环氧氯丙烷相关蛋白1（Kelch-like ECH-associated protein-1, Keap1）途径降低GPX4表达，与顺铂诱导的NSCLC细胞铁死亡协同作用。RSL3还被证明可以通过激活NSCLC细胞中的Nrf2/血红素加氧酶-1（heme oxygenase-1, HO1）通路来抑制GPX4的表达来诱导铁死亡^[57]^。Li等^[58]^证明，铁死亡诱导剂Erastin和索拉非尼通过调节Nrf2或胱氨酸/谷氨酸抗转运蛋白的表达，有效触发具有顺铂耐药特性的NSCLC细胞的铁死亡，导致细胞内脂质ROS过度积累，从而改变NSCLC细胞对顺铂的敏感性。小檗碱与多种铁死亡诱导剂（包括柳氮磺吡啶、RSL3和双氢青蒿素）的组合，通过p53依赖性SLC7A11-GPX4途径耗尽GSH，协同抑制NSCLC^[59]^。目前，GPX4抑制剂作为通过诱导铁死亡杀伤肿瘤细胞的新策略，其临床转化仍处于早期阶段。总体而言，靶向GPX4的铁死亡疗法是一个新兴方向，其最终的临床潜力与转化路径仍有待后续试验数据的验证。

### 4.3 自噬与铁死亡联合治疗的协同效应

鉴于自噬与铁死亡之间存在复杂的作用，联合靶向自噬与铁死亡可能会产生协同抗肿瘤效应。研究^[60]^表明，针对肺癌化疗中细胞凋亡耐药及FIN56诱导铁死亡存在氧化应激不足、易触发防御系统的问题，将FIN56与胡椒胺（哌龙古明）共同封装于丝素蛋白基纳米破坏物FP@SFN中，通过该纳米载体实现二者共递送，其结果显示FP@SFN能有效清除A549细胞、抑制皮下肺癌肿瘤，其主要借助铁死亡-自噬协同机制，增加氧化应激并促进细胞膜破裂，从而增强铁死亡治疗肺癌的效果，为相关疗法开发提供参考。在克服肺癌细胞耐药问题方面，Chen等^[61]^以萘酰亚胺为骨架、通过偶氮键引入特定功能团，开发出可激活荧光前药CFJ001；该前药在癌细胞溶酶体中会生成抗癌小分子氨萘非特（Amonafide）与活性烷基化剂苯胺芥子，对顺铂耐药肺癌细胞的抗增殖活性优于顺铂、阿法替尼等药物，其通过特定的自噬驱动铁死亡机制，促使Fe^2+^积累、细胞膜脂质过氧化及线粒体损伤，最终诱导耐药肺癌细胞铁死亡。目前关于应用治疗肺癌的研究较少，仍需更多深入探索以明确其协同机制并推动临床转化。

### 4.4 自噬相关铁死亡和免疫治疗的潜力

在ICIs，尤其是PD-1/程序性死亡配体1（programmed cell death ligand 1, PD-L1）抗体，已成为多种肿瘤的标准治疗方案，极大地推动了肿瘤免疫治疗的发展^[62]^。通过解除免疫抑制，激活机体免疫系统攻击肿瘤，ICIs在黑色素瘤、NSCLC等多种癌症中取得了显著疗效。然而，免疫治疗的整体有效率较低，大部分患者表现出原发性耐药或获得性耐药，且肿瘤免疫逃逸、免疫抑制性TME是限制治疗效果的主要因素。因此联合疗法被视为改善肿瘤治疗的新途径，其中STAT3抑制剂与免疫疗法的组合便是联合治疗策略的典型范例。氯尼他胺通过抑制STAT3诱导的PD-L1转录，增强了抗PD-1/PD-L1抗体在NSCLC中的疗效^[63]^。一项采用人类癌组织样本（侵袭性乳腺癌、肺癌和宫颈癌）的临床试验^[64]^表明，抗PD-1抗体联合HMGB1抑制剂显著提高了肿瘤细胞死亡率和肿瘤生长抑制率，优于单独使用任一疗法。

然而，联合治疗面临的挑战也不容忽视，包括药物合用带来的毒性管理、免疫相关不良反应（如免疫介导的炎症）以及患者的治疗耐受性。此外，铁死亡和自噬在不同细胞类型和TME中可能产生相反作用，如何精准调控相关信号通路以实现最佳治疗效果仍需深入研究。未来需加强机制研究和临床验证，优化联合方案，解决安全性和耐受性问题，推动其向临床转化。

## 5 总结与展望

本文系统综述了自噬与铁死亡在肺癌发生发展及治疗中的作用与分子机制，揭示了二者通过铁蛋白自噬、线粒体自噬、脂滴自噬、生物钟自噬、CMA等多种选择性自噬途径协同调控肺癌进展的复杂网络，构建了以“自噬-铁死亡”轴为核心、整合ncRNA动态调控与TME免疫重塑的多层次理论框架。现有研究表明，多种天然药物活性成分及纳米递药系统通过靶向自噬-铁死亡轴，可有效诱导肺癌细胞死亡并逆转耐药，展现出多层次的干预潜力。然而，现有研究存在局限，自噬与铁死亡共同作用的具体分子开关尚未明确；当前研究仍较多局限于临床前探索，自噬与铁死亡在TME中的时空动态调控及ncRNA介导的转录后调控网络尚未系统解析，靶向药物的临床转化证据仍显不足。未来研究应致力于在以下方面实现突破：深入解析自噬-铁死亡轴在肺癌不同亚型及TME中的动态调控规律；构建基于ncRNA谱与免疫特征的精准治疗分层体系；推动靶向自噬-铁死亡关键节点的临床前研究向临床试验转化，以期实现从机制到临床的高效路径，为肺癌的防治提供创新性策略。
